# Feasibility and acceptability of at-home play kits for middle school physical activity promotion during the COVID-19 pandemic

**DOI:** 10.1186/s12889-023-15338-y

**Published:** 2023-03-30

**Authors:** Emily Kroshus, Kiana Hafferty, Kimberly Garrett, Ashleigh M. Johnson, Leighla Webb, Andrew Bloom, Erin Sullivan, Kirsten Senturia, Pooja S. Tandon

**Affiliations:** 1 Department of Pediatrics, University of Washington, Seattle, USA; 2 Center for Child Health, Behavior and Development, Seattle Children’s Research Institute, Seattle, USA; 3 School of Exercise and Nutritional Sciences, San Diego State University, San Diego, USA; 4 Upower, Seattle, USA; 5 Seattle Children’s Research Institute, Seattle, USA; 6 School of Public Health, University of Washington, Seattle, WA USA

**Keywords:** Phyiscal activity, School, Pandemic, Play kits, Program development

## Abstract

**Background:**

Schools are central to providing opportunities for youth physical activity (PA), however such opportunities were limited during the COVID-19 pandemic. Identifying feasible, acceptable, and effective approaches for school-based PA promotion amid pandemic-related barriers can inform resource allocation efforts in future circumstances necessitating remote instruction. The aims of this study were to: (1) describe the pragmatic, stakeholder-engaged and theory-informed approach employed to adapt one school’s PA promotion efforts to pandemic restrictions, leading to the creation of at-home “play kits” for students, and (2) assess the feasibility, acceptability, and preliminary effectiveness of this intervention.

**Methods:**

Intervention activities occurred in one middle school (enrollment: 847) located in a Federal Opportunity Zone in the Seattle, WA area, with control data from a nearby middle school (enrollment: 640). Students at the intervention school were eligible to receive a play kit during the quarter they were enrolled in physical education (PE) class. Student surveys were completed across the school year (*n* = 1076), with a primary outcome of days/week that the student engaged in ≥ 60 min of PA. Qualitative interviews (*n* = 25) were conducted with students, staff, parents, and community partners, and focused on play kit acceptability and feasibility.

**Results:**

During remote learning play kits were received by 58% of eligible students. Among students at the intervention school only, students actively enrolled in PE (versus not enrolled) reported significantly more days with ≥ 60 min of PA in the previous week, however the comparison between schools was not statistically significant. In qualitative interviews, most students reported the play kit motivated them to participate in PA, gave them activity ideas, and made virtual PE more enjoyable. Student-reported barriers to using play kits included space (indoors and outdoors), requirements to be quiet at home, necessary but unavailable adult supervision, lack of companions to play outdoors, and inclement weather.

**Conclusions:**

A pre-existing community organization-school partnership lent itself to a rapid response to meet student needs at a time when school staff and resources were highly constrained. The intervention developed through this collaborative response—play kits—has potential to support middle school PA during future pandemics or other conditions that necessitate remote schooling, however modifications to the intervention concept and implementation strategy may be needed to improve reach and effectiveness.

**Supplementary Information:**

The online version contains supplementary material available at 10.1186/s12889-023-15338-y.

## Introduction

Ensuring all youth engage in regular physical activity (PA) is important given its positive impact on both mental and physical health [1]. Prior to the COVID-19 pandemic, fewer than one in four youth in the United States (U.S.) met recommendations of 60 min/day of moderate-to-vigorous intensity PA [2]. During the COVID-19 pandemic, youth PA further declined [3, 4]. Pre-pandemic, schools played a central role in providing children opportunities for PA. The “gold standard” framework for school-based PA promotion is the Comprehensive School Physical Activity Program (CSPAP), [5] which emphasizes the role schools play in providing opportunities for PA during and outside of the school day, and the importance of family and community engagement [5]. School-based PA was particularly challenged during the COVID-19 pandemic as many schools closed for in-person instruction and transitioned to remote learning. A large survey of physical education (PE) teachers in the U.S. documented numerous barriers to remote PE, including lack of accountability, lack of support for program development compatible with online format, technology issues for staff and students, and difficulty meeting the needs of all students including those without internet access [6]. Extracurricular PA opportunities were also limited due to facility closures and restrictions on group gatherings. Identifying feasible, acceptable, and effective approaches for school-based PA promotion amid pandemic-related restrictions and barriers can inform efforts to efficiently allocate resources in future pandemics and during other circumstances that challenge typical school-based PA programming.

Pre-pandemic, community-based Upower and Seattle Children’s Hospital researchers were working closely with one middle school located in a Federal Opportunity Zone, identifying ways to improve access to inclusive and enjoyable PA before, during, and after the school day. Upower is a non-profit organization with a mission to “build positive relationships through trauma-informed coaching, using movement and play to empower and educate youth who are underserved” [7]. When schools closed for in-person instruction, Upower used a pragmatic, stakeholder-engaged and CSPAP-informed approach to adapt their PA promotion efforts to this new reality. This adaptation process, discussed in in subsequent sections, resulted in UHome, a “play kit”-based program for middle school students. Prior studies have found that play kits, comprised of PA supportive equipment and informational resources, are an acceptable intervention for elementary school children [8] with the potential to increase PA [9] as well as skills and attitudes that may be predictive of future increases in PA [10]. Key modifiers of play kit effectiveness among elementary school-aged children include reminders for use (e.g., motivational phone calls) and engagement by caregivers [10].

## Study aims

As Upower worked in partnership with school stakeholders to disseminate play kits, the research team collected formative evaluation data to help refine their approach to intervention and implementation. Guided by the Obesity-Related Behavioral Interventions Trials (ORBIT) Model, [11] we determined that a Phase 1a evaluation was most appropriate. Goals of a Phase 1a evaluation include defining and refining the basic elements of the intervention, and obtaining data related to feasibility and acceptability [11]. Common methods for Phase 1a evaluation studies include qualitative interviews with key stakeholders, and evidentiary studies (“small observational studies of the impact of potential treatment components on process variables”) [11]. Study activities were informed by the RE-AIM (reach, effectiveness, adoption, implementation, maintenance) framework, [12, 13] which has been used to assess the translation of youth physical activity interventions from research to practice [13, 14]. The aims of this study were to: (1) describe the pragmatic, stakeholder-engaged and theory-informed approach employed to adapt one school’s PA promotion efforts to pandemic restrictions, leading to the creation of at-home “play kits” for students, and (2) assess the feasibility, acceptability, and preliminary effectiveness of this intervention.

## Methods

### Sample

The play kit intervention was trialed in one middle school located in a Federal Opportunity Zone in an urban community in the greater Seattle region in the United States. Middle schools in this region serve youth in 6^th^, 7^th^, and 8^th^ grade, typically corresponding to ages 11–14. While the focus of this Phase 1a trial was on feasibility and acceptability of play kits at the intervention school, we also had the opportunity to collect student-level data from a nearby school in the same district that did not receive play kits. All research activities were approved by Seattle Children’s Hospital’s Institutional Review Board (STUDY00002204).

## Procedure for play kit implementation

Students at the intervention school were eligible to receive a play kit in the quarter they were enrolled in PE. For the first two quarters of school year, play kits were distributed to students in tandem with meal pickups, which occurred at the school. A majority of students in the district qualify for free or reduced-price lunch, and during the COVID-19 pandemic, schools continued to provide breakfast and lunch to all students regardless of income status, with pickups several times per week. During the first quarter, play kits were distributed on 3 meal pickup days. In the second quarter, they were distributed on 1 meal pickup day. Based on student and school feedback, Upower, school, and research staff distributed play kits directly to the homes of all 240 students eligible for play kits in the third quarter.

## Procedure for survey data collection

All students enrolled at participating schools were eligible to complete surveys. An information sheet translated into the three languages most frequently spoken by families in the district (English, Spanish, and Vietnamese) was distributed from the school to all parents via school communication platforms. Multiple means for opting children out of the study were provided (i.e., online form, email, telephone number). Subsequently, an information sheet and an assent document were distributed to students whose parents did not opt them out of the study, followed by a web-based questionnaire hosted on the REDCap platform to assenting students. Surveys were completed at the beginning of the school year (September 2020) and then at the end of each of the school quarters when school was held remotely (November 2020, January 2020, March 2020).

## Survey measures

### Physical Activity

Self-reported PA was measured using the single question approach used in the Washington Healthy Youth Survey [15] (“During the past 7 days, on how many days were you physically active for a total of at least 60 min per day? [Add up all the time you spent in any kind of physical activity that increased your heart rate and made you breathe hard some of the time.]”).

### Psychosocial variables

PA enjoyment was assessed with 7 items from the Physical Activity Enjoyment Scale (PACES) [16, 17]. PA self-efficacy was assessed with an 8-item scale and modified for the COVID-19 context. PE enjoyment was assessed using the 4-item Physical Education Enjoyment Scale [18]. All scales used a Likert scale ranging from 1-Disagree a lot to 5-Agree a lot. Internal consistency reliability was acceptable for all three scales (Cronbach’s alpha = 0.93 for PA enjoyment, 0.84 for PA self-efficacy, and 0.94 for PE enjoyment).

### Demographic variables

Grade, self-identified gender, and race and ethnicity (using U.S. Census categories) were captured. Socioeconomic status was assessed using a subset of items from the Family Affluence Scale (FAS) [19] (number of cars owned by family, number of computers owned by family, and does respondent have their own bedroom) based on stakeholder feedback about appropriateness of items from this scale for the current time period and U.S. context. At each survey time point, participants indicated whether they were currently enrolled in PE; at both schools participating in the study all students are required to enroll in PE class for one quarter of the school year.

## Procedure for qualitative data collection

At the intervention school, a purposeful sample of 15 students, 4 middle school staff, 2 district staff, 2 parents, and 2 community partners participated in qualitative interviews. Students were recruited via online flyer, and adult stakeholders were recruited via key informant led snowball sampling. Interviews occurred either over the phone or Zoom (a videoconferencing application). The initial sample estimates achieved data sufficiency, and thus the sample size did not change after an early review of transcripts [20]. Participants provided verbal consent and were compensated $50 each. The research team involved in qualitative aspects of the study included a pediatrician and researcher (PT), a public health researcher (EK), a medical anthropologist (KS), two research scientists (AJ, BB) and three research coordinators (KG, KH, CA). Data were collected by KH, AJ, CA, and analyzed by KH, BB, and AJ, who were trained in qualitative interviewing, codebook development, coding and synthesis by KS who supervised the activities. This report conforms to the Standards for Reporting Qualitative Research [21].

## Qualitative data tools

Interview guides were developed according to study goals and adjusted as necessary throughout data collection as per standard qualitative methodology [22]. Interview questions addressed acceptability and feasibility of play kits from both the student and stakeholder perspective. Additionally, Upower staff provided closed-ended information about play kit distribution (e.g., frequency, number of play kits distributed per distribution time point), and school staff provided information about the number of students eligible to receive play kits. Interviews with students focused on use of, and barriers to, play kits during and beyond the school day. All participants completed short written demographic questionnaires.

## Analysis

### Framework

Consistent with ORBIT Phase 1a evaluation goals, [11] we sought to learn more about the feasibility and acceptability of the play kit intervention and its dissemination and implementation, and to obtain preliminary data related to its potential impact. We were broadly guided by the RE-AIM framework [12, 13]. Adoption and implementation were assessed with program distribution documentation and qualitative data from school staff and Upower personnel, and reach and effectiveness were assessed with survey and qualitative data from students. Given the goals of a Phase 1a trial, maintenance was not assessed.

### Survey data

Demographic data were summarized descriptively using counts and percentages and compared by school (intervention and control) using Chi-Square tests. All participants filling out at least one survey were included in the demographic summary. Scores for PA efficacy, PA enjoyment, and PE enjoyment were calculated using the mean response across questions. Responses with two or more missing responses were excluded. Generalized estimating equations (GEE) models accounting for within subject clustering of responses across time were used to model continuous outcome measures (PA efficacy score, PA enjoyment score, PE enjoyment score, number of days active for 60 min in the previous week). The primary predictor of interest (school participating in intervention vs. control) was modeled with an interaction term to assess whether changes in PA metrics associated with active PE participation differed by school. All models controlled for months elapsed since baseline survey, race, sex, grade, and FAS socioeconomic indicators. As not all questions from the validated survey were collected for calculating FAS score, each of the three questions were included in the model separately. All estimates are presented with 95% confidence intervals and an alpha of 0.05 was used for significance testing. Analyses were conducted using SAS 9.4 (Cary, NC).

## Qualitative analysis

Interviews were digitally recorded, professionally transcribed verbatim, and spot checked by interviewers to ensure data integrity. Every effort was made to maintain participants’ confidentiality during data collection and manuscript preparation. No names are attached to any of the data. In the results, quotes are identified by participant number and type (parent, student, school staff, community partner). Data were uploaded into Dedoose Version 7.0.23 (Sociocultural Research Consultants, Los Angeles, California) for coding and content analysis [23, 24] following the procedures outlined by Braun and Clarke [25]. A hierarchically organized codebook was developed based on the study team's research goals, discussion guide topic areas, and an initial data review. Steps to codebook development were as follows: initial codes were derived from study goals and instrument questions; codes were adapted and augmented by a reading of two transcripts; codes were tested on three additional transcripts by both coders; the codebook was edited as appropriate until an exhaustive but manageable code list was reached. Transcripts were open-coded (KH, BB) using the final version of the codebook. Coders were blind to each other's coding and all differences were resolved by discussion until 100% agreement was reached. When appropriate, the codebook was modified to accommodate new codes or definitions. During synthesis, coded excerpts were systematically annotated and summarized into themes and subthemes with associated quotes.

## Results

### Program development

At onset of the pandemic, Upower leadership met with school stakeholders (teachers, administrators) to identify school priorities, needs, and restrictions related to PA promotion in this changing landscape. As a member of the King County Play Equity Coalition, they also received guidance and support from peers about how other community-based organizations were responding pandemic-related challenges, and assets in the local community that could help this response. Key considerations emerged from this process: the need for all programming and support to be remote; pandemic safety (e.g., social distancing, no group gatherings); challenges with synchronous support/instruction due to technology; school resource and staff time reallocation to pandemic-related priorities; and constraints on student access to resources, space, and time for PA in their home and community. In consideration of these priorities, needs, and restrictions, and informed by the CSPAP framework, Upower and school stakeholders developed program goals: provide students with PA opportunities across the day (i.e., during and outside of school hours); maintain strong connections among students and staff members; and ensure programming is appropriate for youth living in resource-constrained home and neighborhood settings. Additionally, students needed to be able to follow COVID-19 safety guidelines while engaging in PA.

To address these goals, and in consideration of available programmatic resources, play kits were identified as a feasible and appropriate intervention strategy. As one Upower staff member stated,

*We pivoted to offering equipment that kids could use on their own in their home, in their yard, in their local schoolyard, down the street, in their park that they can walk to. Get those kids an opportunity to be able to go outside and play in a social distanced way without adding to their screen time and without coming up against any barriers.*

Upower used PE teacher feedback to adapt the previously trialed elementary school play kit concept [8–10] to: (1) middle school age, (2) pandemic conditions, and (3) low resource home and community settings. To adapt the play kit concept for the middle school age, Upower elicited formative feedback via survey of two middle school PE classes (n = 40 students), with a focus on their preferred play kit content. As a result of the feedback, Upower tried to make materials relevant, engaging, and varied: (1) equipment were offered in multiple variations (e.g., students could select from several different types of balls); (2) pandemic-appropriate equipment could be used by individuals (i.e., not requiring a partner); (3) group activity suggestions allowed for social distancing; (4) activities required minimal space and no additional equipment beyond the play kit item (e.g., resistance bands and jump ropes).

### Program description

Play kits included: choice of ball (basketball, football, volleyball, playground ball, or soccer ball), jump rope, resistance band, and activity sheets with ideas for PA before/after school and as an individual or with family. Additional items to help address additional student needs included: masks and hand sanitizer, a water bottle, notebook, apple, and a handout with information about nutrition and social-emotional wellness. Guided broadly by the CSPAP framework, play kit items were intended to support PA during PE (e.g. resistance bands that PE teachers included in their remote PE activities), and outside the virtual school day (e.g., after school, potentially with family members).

### Sample characteristics

Seven hundred fifty intervention school students and 326 control school students completed at least one survey, with school-level response rates of 76% (intervention) and 48% (control). Survey participation was relatively evenly distributed between male (45.3%) and female students (43.1%) and across 6^th^ (32.4%), 7^th^ (30.7%) and 8^th^ grades (36.8%). Additional student descriptive characteristics are provided in Table 1. Participants in qualitative interviews included 15 middle school children grades 6-8^th^ and 8 school stakeholders (teachers, administrators, district PE specialist) and 2 parents. Sample characteristics for qualitative interview participants are provided in the electronic supplement.

**Table 1:** Baseline demographic characteristics of the study population^a^

	Intervention *n* = 750 n (%)	Control *n* = 326 n (%)	*p*-value
Grade^b^			
6th	243 (32.4%)	118 (36.2%)	0.086
7th	230 (30.7%)	110 (33.7%)	
8th	276 (36.8%)	97 (29.8%)	
Sex			
Male	340 (45.3%)	135 (41.4%)	0.470
Female	323 (43.1%)	154 (47.2%)	
Non-binary	9 (1.2%)	6 (1.8%)	
Prefer not to answer/Unknown	78 (10.4%)	31 (9.5%)	
Race/Ethnicity			
American Indian/Alaska Native	10 (1.3%)	4 (1.2%)	< 0.001
Asian	119 (15.9%)	30 (9.2%)	
Black/African American	64 (8.5%)	18 (5.5%)	
Native Hawaiian/Pacific Islander	22 (2.9%)	7 (2.1%)	
White/Caucasian	66 (8.8%)	134 (41.1%)	
Hispanic	368 (49.1%)	63 (19.3%)	
More than 1 race	39 (5.2%)	42 (12.9%)	
Unknown	62 (8.3%)	28 (8.6%)	
Does your family own a car, van, or truck?			
Yes, two or more	496 (66.1%)	239 (73.3%)	0.102
Yes, one	175 (23.3%)	59 (18.1%)	
No	21 (2.8%)	5 (1.5%)	
Unknown	58 (7.7%)	23 (7.1%)	
Do you have your own bedroom for yourself?			
Yes	345 (46.0%)	214 (65.6%)	< 0.001
No	341 (45.5%)	88 (27.0%)	
Unknown	64 (8.5%)	24 (7.4%)	
How many computers does your family own?			
None	104 (13.9%)	18 (5.5%)	< 0.001
One	200 (26.7%)	58 (17.8%)	
Two	169 (22.5%)	64 (19.6%)	
More than Two	215 (28.7%)	163 (50.0%)	
Unknown	62 (8.3%)	23 (7.1%)	

^a^Includes all participants who filled out at least 1 survey (first survey) prior to 4/14/2021 ^b^Not answered by 2 participants

### Adoption and reach

Over the three quarters of the school year during which learning occurred remotely, play kits were received by 58% of the 740 eligible students. Upower attended three meal pickups in the first quarter to distribute play kits, with 99 students received play kits (43% of eligible students). Based on school district feedback, Upower only attended one meal pick-up in the second quarter, distributing play kits to 59 students (26% of eligible students). Given the low reach of play kits in the first two quarters, Upower, teachers and research staff distributed play kits directly to the homes of all 240 students eligible for play kits in the third quarter; this means of distribution was preferred by school and district administration.

Qualitative data from school administrators and teachers indicated that they responded positively to play kits, appreciating that play kits gave students choices and had the potential to help increase PA. They also believed the play kits showed students that the school wanted them to be healthy and happy, and that the school cared for families not able to afford equipment. This sentiment was echoed by students in qualitative interviews, who described how receiving the play kit made them feel like their school cared about them. Lastly, school administrators and teachers appreciated that Upower personnel collaborated with school staff and incorporated their input when deciding play kit content.

### Effectiveness

At the intervention school, students were active for at least 60 minutes an average of 3.14 days per week (95% CI = 2.75 to 3.53) when they were not enrolled in PE, and 3.60 days (95% CI = 3.21 to 4.00) when they were enrolled in PE. At the control school, on students were active for at least 60 minutes an average of 3.25 days when not enrolled in PE (95% CI = 2.79 to 3.70), and 3.46 days when enrolled in PE (95% CI = 2.96 to 3.95). Among students at the intervention school, but not among students at the control school, those actively enrolled in PE reported significantly higher levels of PA (number of days active 60 min or more in previous week compared to those not actively enrolled in PE). Adjusting for race/ethnicity, sex, and grade and socioeconomic status (Table 2) this difference was not significantly greater than the difference between those enrolled and not enrolled in PE at the control school. In both the intervention and control school, there was no difference over time in PA efficacy, enjoyment, PE enjoyment, or level of PA. Detail is provided in Table 2.

**Table 2:** Differences in the least squares means of physical activity metrics by active Physical Education (PE) enrollment and school

	Estimate (95% CI)^a^	*p*-value
Physical Activity Efficacy Score		
Enrolled in PE (Intervention)	0.01 (-0.08 to 0.10)	0.863
Enrolled in PE (Control)	-0.01 (-0.19 to 0.17)	0.941
Difference in Slopes (Intervention vs. Control)	0.01 (-0.19 to 0.21)	0.884
Physical Activity Enjoyment Score^b^		
Enrolled in PE (Intervention)	-0.01 (-0.13 to 0.11)	0.840
Enrolled in PE (Control)	0.10 (-0.12 to 0.31)	0.385
Difference in Slopes (Intervention vs. Control)	-0.11 (-0.35 to 0.14)	0.385
PE Enjoyment Score		
Enrolled in PE (Intervention)	0.03 (-0.10 to 0.16)	0.608
Enrolled in PE (Control)	0.07 (-0.16 to 0.31)	0.525
Difference in Slopes (Intervention vs. Control)	-0.04 (-0.30 to 0.22)	0.757
Number of Days in Previous Week Active for 60 min or more		
Enrolled in PE (Intervention)	0.45 (0.17 to 0.73)	0.002
Enrolled in PE (Control)	0.21 (-0.25 to 0.68)	0.369
Difference in Slopes (Intervention vs. Control)	0.24 (-0.29 to 0.77)	0.379

^a^Generalized estimating equations model adjusted for repeated observations within participants as well as months since baseline, race/ethnicity, sex, and grade and socioeconomic status indicators; reference group are those not currently enrolled in PE at the time of survey

^b^Higher scores indicate less enjoyment

Qualitative interview participants provided context about the impact of play kits on PE experiences. Students reported different perceptions: several students reported liking virtual PE class more after receiving a play kit, and some said play kit equipment made PE more enjoyable by making it more challenging, interesting, and more like in-person PE. Most students reported the play kit motivated them to participate in PA and gave them ideas for games/activities. However, students mentioned that participation in PE was not enforced or easily monitored. Additionally, a few students mentioned that they simply did not like exercising with the items they received in the play kit including two students who reported avoiding the resistance band because they hit themselves in the face with it. School PE teachers stated that the play kit did not seem to change student engagement in PE nor make their job easier or harder although students were excited to have a play kit and showed teachers the equipment they received. Additional qualitative results are presented in Table 3.

**Table 3:** Qualitative themes mapped onto RE-AIM (Reach, Effectiveness, Adoption, Implementation, and Maintenance) framework

RE-AIM component	Theme	Example quote
Reach	Distribution	*…acquiring the amount of equipment necessary, finding a place to put it and transporting it … It’s just a lot of stuff, and we don’t have a truck or anything. It’s just all of our staff cars. [103_community partner]*
	Staffing	*Staff were very involved and helped as much as we could, but then our partnership… they helped everywhere they could. … it made it as seamless as possible. [103_community partner]*
	Acceptability to students	*I think the play kits [are] very helpful, especially because we’re in quarantine right now and not a lot of families have supplies for working out or PE at home, or money to buy it. [5 _student]*
Effectiveness	Perceived impact on physical education enjoyment	*All of them wanted to show me the basketball or the football. … they were excited about it, yes. Did that make them more engaged in class? Not necessarily. But they were excited they got the equipment* *[107_school staff]* *Being in PE and having these new items, makes me more excited to exercise more. [234 _student]*
	Perceived impact on physical activity	*We got a ton of just really positive feedback of just general gratitude and appreciation… a grandparent that said that their grandson had, since COVID, just not been doing anything physically active and had nothing to do physically active at home, so getting a ball and a jump rope was gonna be huge because she thought he would really, really use these. [103_community partner]*
Adoption	Acceptability to school staff	*I'm thinking of our students and how often do they get new things?…. So, just that feeling also of knowing that there’s people out there that want to give us things in order to be successful or be healthier, I'm sure that’s also very good for our students to know. [102_school staff]*
Implementation	Planning (school)	*Yeah. I'm honest. I don't know how helpful they were. I don't know how much kids used those. …So, that’s been probably one thing that would be nice if I had so that I could kind of look at to incorporate what that is. [107_school staff]*
	Space/setting (student)	*There’s a jump rope and the basketball. So, that was good. But we also live in an apartment. So, it’s really hard to go and do that. And we live on the top floor. So, you don’t wanna make too much noise. [8 parent]*
	Supervision (student)	*I'll just have to convince my family to go with me because our closest park is a couple blocks away. [102 _student]*

### Implementation

Not all students enrolled in PE during the first two quarters received play kits, so teachers had to adapt lesson plans to accommodate students without play kits. Additionally, some PE teachers did not have access to the play kit when creating their lesson plan for the quarter, making it more difficult to incorporate it into their curriculum.

Students participating in qualitative interviews provided detail on the items they recalled using (Supplementary Table 1). Balls (*n* = 14), jump ropes (*n* = 11), and resistance bands (*n* = 10), were the items most frequently recalled. There were no clear patterns for how items were used or with whom, except that resistance bands were typically used inside. Student-reported barriers to using play kits included small apartments, family at home, requirements to be quiet for neighbors, nowhere nearby outside for play, necessary but unavailable adult supervision, lack of companions to play outdoors, and inclement weather.

## Discussion

Schools play a central role in providing equitable opportunities for youth PA, and when schools are closed and all learning occurs remotely, school-based PA promotion is particularly challenging. To address this problem, distribution of play kits with PA equipment was trialed as a strategy for pandemic PA promotion among middle school students in an economically disadvantaged community. This Phase 1a mixed methods evaluation provides equivocal data about the acceptability, feasibility, and potential for effectiveness of play kits, and suggests ways in which adaptations may be warranted before progressing to a larger pilot evaluation.

The participating school and community-based organization Upower worked together to rapidly develop and implement the play kit intervention. Community partnerships are a core component of the CSPAP model, and in this case the school partnership with Upower functioned to make the play kit intervention feasible during a year with heightened demands on school staff and resources. Prior research finds that school staffing is a primary constraint on CSPAP-informed interventions [26, 27]. In this case, adoption and implementation at the school-level was facilitated by the majority of labor provided by Upower with limited demands placed on the school. For example, based on mid-year process data that not all eligible students had received play kits, the distribution method was modified to home delivery largely by Upower staff. Although engaging community partners can help schools meet student needs, these partners face their own resource constraints. Upower staff time was offset by ongoing grant funding, which can be difficult for small organizations to obtain especially with timelines conducive to a quick turnaround response to events such as the COVID-19 pandemic. In the present context, the school and Upower were able to respond to pandemic conditions and school needs because of pre-existing funding and a funder (Department of Health and Human Services) that encouraged a flexible and community-led approach to adapting programming and research aims to pandemic conditions. Additionally, facilitated in part via the network of the King County Play Equity Coalition, Upower worked with corporate sponsors to obtain sports equipment for free or at reduced cost. While such funder and corporate relationships are not explicitly included in the CSPAP framework, they were critical for program feasibility and sustainability. We encourage others developing and evaluating CSPAP-based PA interventions in schools to articulate the external sources of support used (e.g., financial, staffing), and the implications those have for the conditions under which the program may be sustainable. Collaborative peer networks such as the King County Play Equity Coalition have the potential to help address problems related to program support by connecting community-based programs with existing local resources (financial, tangible, and informational).

Play kits were distributed to students enrolled in PE, and among students at the intervention school there was slightly less than half a day increase in the average number of days per week that students engaged in at least 60 min of moderate to vigorous physical activity. We note that although there was no increase in PA observed at the control school, the difference between intervention and control school was not significant. The difference between schools is difficult to interpret given the substantially lower response rate at the control school—raising questions about the comparability of students. Considering only the experience of the intervention school, we are unable to tease apart the contribution of the play kits to student PA during PE. However, findings do lend support to the important role that remote PE class played in student PA during the pandemic and suggest the possibility that play kits enhanced student PA during remote PE. Further, we note that at the intervention school only 58% of students received play kits, in part due to the evolving strategy for play kit distribution. Individuals who did not pick up play kits (when they were distributed on site at school rather than delivered to their home) may have been systematically different than individuals who sought out play kits. For example, it is possible they were more motivated for PA, or that they differed in terms of their socioeconomic status. Future efforts to test the effectiveness of play kits should build from these findings, using a means of play kit distribution that results in more widespread reach.

We note that play kit intervention content was focused solely on students, and PE teachers expressed some challenges incorporating play kit items into preexisting curriculum. In future iterations of the intervention, it may be useful to provide a companion resource for PE teachers about ways in which play kit equipment can be incorporated into PE activities. Such efforts should engage PE teachers in helping identify items that will support their curricular goals, while also considering student preferences. Furthermore, broader and earlier dissemination of the play kits (not just during the quarter the student was enrolled in PE) may have been more effective in promoting student PA across the school year.

Play kit content was designed to be usable by students alone or with others including family members, however family members were not engaged explicitly in the intervention. Other CSPAP-informed interventions have engaged families through strategies including sending home newsletters or information to families, formal homework assignments that family members complete together, and in some cases health fairs or “family fun nights” [28]. Further research is needed to understand the extent of co-participation in PA in this age group and how to design activities and provide equipment that supports family PA. This may be particularly helpful in supporting use of the play kit outside of PE class.

## Limitations

Students completing surveys at the intervention and control middle schools differed significantly with respect to race and ethnicity and age-relevant indicators of socioeconomic status. Any changes observed may be due to differences in schools at baseline, as we cannot fully account for these differences in the model. All data were collected by self-report and are subject to recall and social desirability biases. Surveys were optional to complete, and there may be bias in which students chose to complete the surveys throughout the school year. Additionally, data were not reliably collected on when each participant received a play kit, so we could not account for whether the students had received one at the time of survey completion.

## Conclusion

This study provides an example of how a pre-existing community organization-school partnership lent itself to a rapid and collaborative response to meet student PA needs at a time when school staff and resources were highly constrained. The intervention developed through this collaborative response—play kits—has potential to support middle school PA during future pandemics or other conditions that necessitate remote schooling. However, modifications to the intervention concept and implementation strategy may be needed. Key considerations include identifying how to better support PE teachers in integrating play kit equipment into virtual PE, considering non-PE opportunities for play kit use, and identifying ways to strengthen family engagement in PA co-participation using play kit equipment. Additionally, the resource demands of distributing a physical product to students who are not all gathering in person is an implementation barrier that must be weighed when considering the benefits of a play kit-based approach to intervention.

## Supplementary Information

**Additional file 1: Supplementary Tables 1 and 2**. 

## Data Availability

Upon reasonable request to corresponding author.
