# Supplementary material for: Feasibility and acceptability of at-home play kits for middle school physical activity promotion during the COVID-19 pandemic
Source: BMC Public Health. 2023 Mar 30;23:604. doi: 10.1186/s12889-023-15338-y (PMC10061390; doi:10.1186/s12889-023-15338-y)
Supplement: Supplementary file 1 — Additional file 1: Supplementary Tables 1 and 2. [file 12889_2023_15338_MOESM1_ESM.docx]

**Supplementary Table 1.** Qualitative interview student participant recall of play kit items (n=15)

| **Item** | **Times recalled**  **n (%)** |
| --- | --- |
| Ball of choice | 14 (93.3) |
| Jump rope | 11 (73.3) |
| Resistance band | 10 (66.7) |
| Activity sheets | 4 (26.7) |
| Nutrition information | 1 (6.7) |
| Social emotional | 0 (0.0)) |
| Apple | 1 (6.7) |
| Water bottle | 8 (53.3) |
| Baseball bases | 2 (13.3) |
| Upower bracelet | 1 (6.7) |
| Hat | 2 (13.3) |
| Mask | 2 (13.3) |
| Hand sanitizer | 1 (6.7) |
| Notebook | 8 (53.3) |
| Do not remember | 1 (6.7) |

**Supplementary Table 2*.*** Qualitative interview student participant recall of how items were used (n=15)

| **Item** | **n** | **Who used it** | **n** | **Where** | **n** | **Still using?** | **n** | **Used for physical education (PE)?** |
| --- | --- | --- | --- | --- | --- | --- | --- | --- |
| Ball | 5 | Alone | 3 | Yard | 4 | Yes | 1 | Yes, in person |
|  | 4 | With siblings | 1 | School (PE) | 4 | 1-2x total |  |  |
|  | 2 | With parent | 3 | Inside | 2 | Monthly |  |  |
|  | 4 | Whole family | 6 | Park | 3 | No |  |  |
| Resistance band | 12 | Alone | 9 | Inside home | 1 | Monthly | 2 | PE Log |
|  | 3 | Family | 3 | School (PE) | 4 | 1-2x / week | 1 | In-person |
|  |  |  |  |  | 2 | 1-2x total |  |  |
|  |  |  |  |  | 2 | 5x per week |  |  |
| Jump rope | 6 | Alone | 5 | Inside | 4 | No | 1 | PE Log |
|  | 3 | Parent | 4 | Yard | 1 | Yes |  |  |
|  | 3 | Siblings | 1 | Park | 3 | 1-2 x total |  |  |
|  |  |  | 2 | Balcony apartment | 1 | Monthly |  |  |
|  |  |  |  |  | 3 | Often at first, but not anymore |  |  |
|  |  |  |  |  | 1 | Almost every day |  |  |
| Activity sheet & Mindfulness | 3 | Alone | 3 | Home | 6 | 1-2 x total |  |  |
|  | 1 | Family | 1 | Park | 8 | Never used |  |  |
| Water bottle |  |  |  |  | 6 | Yes |  |  |
|  |  |  |  |  | 4 | Never |  |  |
| Nutrition |  |  |  |  | 3 | Yes |  |  |
|  |  |  |  |  | 3 | Never |  |  |
| Mask & Hand Sanitizer | 4 | Family |  |  | 5 | Yes |  |  |
|  |  |  |  |  | 2 | No |  |  |
| Notepad |  |  |  |  | 4 | Yes | 1 | PE class |
|  |  |  |  |  | 2 | No |  |  |
